# SHBG and total testosterone levels in men with adult onset hypogonadism: what are we overlooking?

**DOI:** 10.1186/s40842-020-00106-3

**Published:** 2020-09-29

**Authors:** Stephen J. Winters

**Affiliations:** Division of Endocrinology, Metabolism and Diabetes, University of Louisville, ACB-A3G11, 550 Jackson Street, Louisville, KY 40202 USA

**Keywords:** SHBG, Testosterone, Male hypogonadism, Metabolic syndrome

## Abstract

**Background:**

Adult onset male hypogonadism (AOH) is a common clinical condition whose diagnosis and management are controversial, and is often characterized by a low level of SHBG, but our understanding of why testosterone levels are low when SHBG is low is incomplete.

**Methods:**

This retrospective chart review was performed to compare the relationship between SHBG and testosterone in the plasma of men presenting for evaluation of AOH with a cohort of men treated chronically with transdermal testosterone.

**Results:**

The level of SHBG was < 30 nmol/L in 73% of men who presented for evaluation of AOH, and was inversely proportional to BMI in both the untreated and the testosterone-treated men. As in previous populations, the level of SHBG was highly positively correlated (*r* = 0.71, *p* < 0.01) with the total testosterone level in untreated men presenting for evaluation of AOH, but no relationship was found between the level of SHBG and total testosterone among men who were being treated with a transdermal testosterone preparation.

**Conclusions:**

These findings further support the idea that SHBG regulates testicular negative feedback either directly or by modulating the entry of testosterone or estradiol into cells in the hypothalamus and/or pituitary to control gonadotropin synthesis and secretion which explains in part the low testosterone levels in men with AOH.

**Trial registration:**

Not applicable

## Background

Adult onset male hypogonadism (AOH) is a clinical condition characterized by symptoms consistent with androgen deficiency together with a consistently low total testosterone level that is not explained by classical disorders of the hypothalamus-pituitary or the testis [1]. Its diagnosis and management remain controversial [2, 3]. Many men given this diagnosis have a low level of sex hormone-binding globulin (SHBG) associated with obesity, type 2 diabetes (T2DM), dyslipidemia and non-alcoholic fatty liver disease (NAFLD).

SHBG is a homodimeric glycoprotein, and both monomers can bind sex steroids [4, 5]. The binding affinity of SHBG for testosterone is high, and has been reported to vary across total testosterone levels [6], and to demonstrate allosteric interaction between the two binding sites [7]. Thus, it follows that the level of SHBG, which binds testosterone with high affinity and transports testosterone in the circulation, is strongly positively correlated with the level of testosterone in plasma [8, 9].

What is unclear is whether the low testosterone level in men with AOH is due solely to less circulating SHBG or whether additional mechanisms are involved. A long-standing idea is that increased estradiol production in AOH suppresses GnRH-LH secretion and testosterone biosynthesis. In support of this idea, many men with AOH are overweight, aromatase is expressed in subcutaneous adipose tissue [10], and estradiol levels are often increased in obese men [11, 12]. However, not all data support this notion. For example, estradiol levels did not correlate inversely with testosterone with increasing obesity [13]. Moreover, total and calculated free estradiol levels were lower, rather than higher, among men with T2DM with low total and calculated free testosterone levels, when compared to values among diabetic men with normal total and free testosterone [14]. Furthermore, in the European Male Aging Study, men diagnosed with late onset hypogonadism, with lower testosterone and SHBG levels and a higher waist circumference and BMI than the eugonadal men, also had lower estradiol levels [15]. A second idea is that elevated pro-inflammatory cytokines, such as TNFα and interleukins, suppress GnRH and testicular steroidogenesis. In this regard, when interleukin-2 was administered iv to men, testosterone levels declined but LH levels were unchanged [16]. Third, leptin activates kisspeptin neurons to stimulate GnRH [17], and leptin deficiency is associated with hypogonadotropic hypogonadism [18]. Moreover, leptin treatment restores the testosterone deficiency that occurs with fasting [19]. As obese individuals are viewed as resistant to leptin, leptin resistance may also play a role in AOH.

Another possible explanation is that variation in SHBG, either directly or through free testosterone and/or estradiol levels, regulates GnRH-LH and thereby testosterone and estradiol secretion until a new equilibrium is reached [20, 21]. In support of this idea, deRonde et al. [22] found a positive association between SHBG and testosterone in newborn boys in whom the GnRH pulse generator is active, as in adult men, but this relationship is absent among prepubertal boys age 5–8 yrs [23] whose GnRH pulse generator is suppressed, and is negligible in umbilical cord blood [24]. Furthermore, SHBG levels are inversely related to testosterone in normal pre- and post-menopausal women [25] among whom testosterone is not viewed as an important regulator of GnRH-LH secretion. If SHBG regulates LH secretion and contributes to the strong positive correlation between circulating SHBG and testosterone levels in men, the relationship should be present in untreated men with AOH whose hypothalamic-pituitary-testis is functional, but may be attenuated or absent in testosterone –treated men. This retrospective analysis was conducted in order to explore this hypothesis.

## Methods

Medical records were reviewed retrospectively from adult men who presented for a clinical evaluation of adult onset hypogonadism (*n* = 33), and from adult men who were being treated for hypogonadism with transdermal testosterone (*n* = 25).

The men with AOH presented for evaluation because of erectile dysfunction, low libido and/or asthenia. A thorough medical history and physical examination were performed, and classical causes of hypogonadism were excluded, in most cases by additional endocrine testing. Testis size was ≥ 20 mL. 6 men had diabetes, and 16 were being treated for dyslipidemia.

Testosterone-treated men had been diagnosed with adult onset hypogonadism (*n* = 12), hypopituitarism (*n* = 9), congenital hypogonadotropic hypogonadism (*n* = 2), or Klinefelter syndrome (*n* = 2), and were being treated with a stable dose of a transdermal testosterone preparation.

Assays were performed at Quest Diagnostics. Testosterone levels were measured by liquid chromatography-mass spectrometry, and SHBG was measured by immunoassay. Free testosterone was measured by indirect equilibrium dialysis. Laboratory reference ranges for adult men are: 250–1100 ng/dL for total testosterone, 10–50 nmol/L for SHBG, and 35–155 pg/mL for free testosterone.

### Statistical analysis

Two group comparisons were performed using Student’s t-test or the Mann–Whitney Rank Sum test when data were not normally distributed or groups had unequal variance. Pearson correlation coefficients were calculated using SigmaStat/Systat Software, Inc. (San Jose, CA). Data are presented as the mean ± SD or the median and 25–75% range.

## Results

The characteristics of the patient groups are summarized in Table 1. Of the 33 men seeking an evaluation for hypogonadism, 7 men had a total testosterone level below the assay reference range (< 250 ng/dL, 8.68 nmol/L) and 14 were below 300 ng/dL (10.4 nmol/L), a level which is often selected as the threshold to diagnose testosterone deficiency [26]. Only one man had a free testosterone level below the reference range (35–155 pg/ml). Testosterone-treated men were older (*p* = 0.002) and had a lower BMI (*p* = 0.04) than the men with AOH. Among the testosterone- treated men, total testosterone levels were within the reference range in 22/24.

**Table 1:** Clinical characteristics for men presenting for evaluation and treatment of adult onset hypogonadism, and men treated chronically with transdermal testosterone

	Adult onset hypogonadism (*n* = 33)	T-treated hypogonadal men (*n* = 25)	*P* value
Age (yrs)	45 (31.5–55.0)^a^	61.0 (48.0–67.5)	.002
BMI (kg/m^2^)	32.0 (27.8–39.3)	29.1 (24.6–33.1)	.04
Testosterone (ng/dL)	317 (237–391)	452 (360–528)	.001
SHBG (nmol/L)	25.7 (21.2–35.0)	31.0 (22.5–40.5)	.189
Free testosterone (pg/mL)	50.7 (44.5–61.5)	65.3 (45.8–95.0)	.084

To convert total testosterone levels to nmol/L multiply by 0.0347

^a^Median (25–75%)

As summarized in Table 2, those men in the AOH cohort with a total testosterone level < 300 ng/dL were similar in age to those with a normal total testosterone concentration, but had a higher BMI (*p* = 0.002) and a lower level of SHBG (*p* = 0.002). SHBG was < 20 nmol/L in 9 men, and was < 30 nmol/L in 24 of the men (73%) who presented for evaluation of AOH.

**Table 2:** Characteristics of men presenting for evaluation and management of AOH

	Low testosterone (*n* = 14)	Normal testosterone (*n* = 19)	*P* value
Testosterone (ng/dL)	253 (191–285)^a^	350 (320–439)	.001
Age (yrs)	42.0 (33.8–56.0)	45.0 (29.0–53.0)	.72
BMI (kg/m^2^)	37.4 ± 7.1	30.2 ± 5.8	.002
SHBG (nmol/L)	21.5 (12.7–25.0)	33.0 (24.0–42.0)	.002
Free testosterone (pg/mL)	48.6 ± 13.7	57.2 ± 13.9	.11

To convert total testosterone levels to nmol/L multiply by 0.0347

^a^Median (25–75%) or mean ± SD

**Fig. 1:**
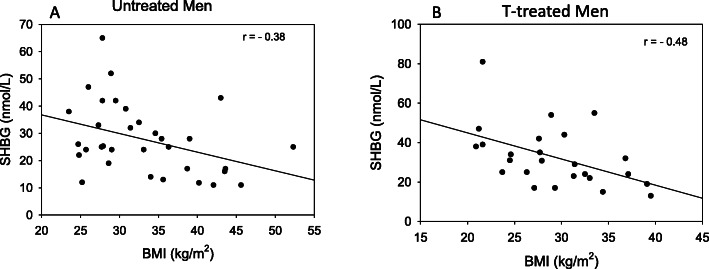
Relationship between BMI and SHBG in untreated men who presented for evaluation of AOH (**a**) and a cohort of men who were being treated with a transdermal testosterone preparation (**b**). In **a**) BMI = 38.808—(0.206 * SHBG; *r* = -0.38, *p* = 0.032, and **b**) BMI = 33.352—(0.0755 * SHBG); *r* = -0.48, *p* = 0.05

**Fig. 2:**
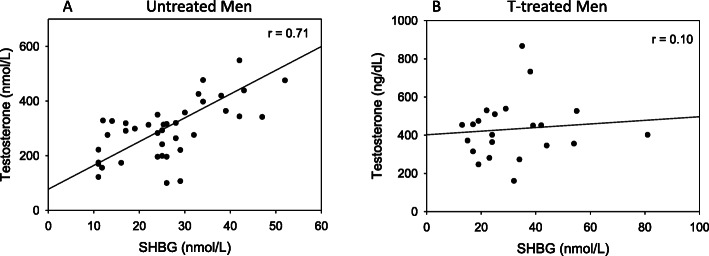
Relationship between the plasma levels of SHBG and total testosterone among men presenting for evaluation of AOH (**a**) and a cohort of men who were being treated with a transdermal testosterone preparation (**b**). In **a**, testosterone = 77.532 + (8.709 * SHBG), *r* = 0.71, *p* < 0.01, and in **b**) testosterone = 371.209 + (2.023 * SHBG); *r* = 0.1, *p* = 0.16

The level of SHBG was inversely proportional to BMI in both the untreated and the testosterone-treated men (Fig. 1). The relationship between SHBG and total testosterone is shown for the untreated and T-treated men in Fig. 2. As in previous analyses of results in eugonadal men [8, 9, 22], the level of SHBG is highly positively correlated (*r* = 0.71, *p* < 0.01) with the total testosterone level in untreated men who presented for evaluation of AOH. On the other hand, there is no significant relationship between the levels of SHBG and total testosterone among men who were being treated with a transdermal testosterone preparation (Fig. 2).

## Discussion

The findings of this study provide further support for the idea that the mechanism for the strong positive correlation between the circulating levels of SHBG and total testosterone in men is more complex than the stoichiometry of a high affinity circulating transport protein which controls metabolic clearance, and its ligand. Instead, the positive correlation in untreated, but not in testosterone-treated men, implies that SHBG regulates testicular negative feedback either directly or by modulating the entry of testosterone or estradiol into cells in the hypothalamus and/or pituitary to control gonadotropin synthesis and secretion, and thereby testosterone levels.

An effect of SHBG on gonadotropin and sex steroid secretion is implied from observations in men with hyperthyroidism who typically have high serum levels SHBG as well as LH and testosterone [27, 28]. Likewise, SHBG as well as LH and testosterone levels increased when normal men were rendered hyperthyroid by thyroxine administration [29], and normalize when hyperthyroid men become euthyroid [28]. These findings are likely explained by the effect of thyroxine to increase SHBG mRNA levels by stimulating the transcription factor hepatocyte nuclear factor 4α (HNF4α) [30] that activates the SHBG promoter [31].

The concept that a circulating high affinity binding protein regulates hypothalamic-pituitary function also follows from the changes that occur in hypothyroid women who become pregnant. During pregnancy, there is a twofold increase in circulating thyroglobulin levels due to the effect of increased placental estrogens [32, 33]. TSH levels rise, but thyroid dysfunction prevents the normal compensatory increase in thyroid hormone synthesis, and TSH remains elevated. Therefore, the thyroxine dose is generally increased in hypothyroid women during pregnancy.

Despite some inconsistent findings, estradiol negative feedback remains an attractive potential explanation for the low testosterone level in AOH. Many studies have shown that much of the testicular negative feedback control of gonadotropin secretion in men is through estradiol. When estradiol is infused iv at physiological levels, LH is suppressed [34, 35]. SERMs with antiestrogenic effects in the CNS increase circulating LH as well as testosterone levels in normal men [36, 37], as do aromatase inhibitors [38, 39]. Furthermore, men with inactivating mutation of the aromatase gene often have high LH and testosterone levels [40]. The measurement approaches for free and/or non-SHBG testosterone levels continue to be debated [41, 42], and quantification of non-SHBG-bound estradiol is even more challenging. Total estradiol levels in normal men are < 1% of total testosterone levels, and accurate measurement requires LC–MS methodology [43]. For free estradiol, even direct assay by LC–MS following dialysis of the plasma sample will be problematic since the value may be 2–3% [44] of 20–30 pg/mL, or < 1 pg/mL. Results of previous studies in which free or bioavailable estradiol levels were determined by immunoassays or by calculation, and were related to testosterone in AOH and other outcomes, will need to be confirmed.

SHBG levels are often decreased in men who present for evaluation of AOH [45, 46]. In this study, 13/14 untreated men with a testosterone level of < 300 ng/dL had an SHBG level of < 30 nmol/L, and increased BMI predicted lower SHBG levels in not only untreated but also testosterone-treated men. These observations support the importance of metabolic regulation of SHBG, and underscore the need to measure SHBG in the clinical evaluation of men with suspected AOH in order to identify those who are at increased risk for Metabolic Syndrome, T2DM, NAFLD and cardiovascular disease.

This is a provocative but limited study. Limitations include a relatively small sample size, and not all T-treated men had AOH. Only one blood sample was obtained. Finally, the cross-sectional analysis does not provide further insight into why some adult men develop low SHBG levels. These observations may, however, encourage a rethinking and further studies into the link between SHBG and testosterone in AOH.

## Conclusions

The level of SHBG is low in most men presenting for evaluation of AOH. There is a strong positive correlation between the levels of SHBG and total testosterone among untreated men with AOH but not in testosterone-treated men. This finding implies that SHBG regulates testosterone production through a negative feedback mechanism either directly or by modulating the entry of testosterone or estradiol into cells in the hypothalamus and/or pituitary. Measuring SHBG is helpful in understanding the pathophysiology and in management decisions for men with AOH.

## Data Availability

Data are available upon request.

## Abbreviations

AOH: Adult onset hypogonadism
BMI: Body mass index
LC–MS: Liquid chromatography-mass spectroscopy
LH: Luteinizing hormone
SERM: Selective estrogen response modifiers
NAFLD: Non-alcoholic fatty liver disease
T2DM: Type-2 diabetes mellitus
SHBG: Sex hormone-binding globulin
T: Testosterone
TSH: Thyroid stimulating hormone
